# Supplementary material for: Mechanical Detuning of Exciton–Phonon Resonance in WS2
Source: ACS Photonics. 2026 Mar 24;13(8):2199–205. doi: 10.1021/acsphotonics.5c03089 (PMC13088364; doi:10.1021/acsphotonics.5c03089)
Supplement: Supplementary file 1 [file ph5c03089_si_001.pdf]

Supporting Information for:

Mechanical Detuning of Exciton-Phonon Resonance in WS_2

Álvaro Rodríguez[1*], Carmen Munuera[1], Andres Castellanos-Gomez[1]

[1]Instituto de Ciencia de Materiales de Madrid (ICMM-CSIC), C. Sor Juana Inés de la Cruz, 3, Madrid, 28049, Spain.

*Corresponding author(s): alvaro.rodriguez@csic.es

Table of contents:

S1. Experimental section

S2. Biaxial straining setup

S3. Identification of number of layers

S4. Raman fits for monolayer and bilayer WS_2

S5. Background subtraction procedure for ΔR/R spectra

S6. Spatial uniformity of the applied biaxial strain

S7. Reversibility and absence of hysteresis under biaxial strain

S8. Double-resonant Raman model and effective detuning description

S1. Experimental section

Sample Fabrication. WS_2 (HQ Graphene) was exfoliated using Nitto tape onto 250 μm thick polycarbonate substrates (Modulor GmbH) previously cut into a cross-shaped geometry. The PC pieces were coated with 3 nm Ti and 6 nm Au using a home-built electron-beam

evaporator. Gold-assisted exfoliation enabled the preparation of large-area WS_2 monolayers (predominantly), as well as bilayer, trilayer and few-layer flakes.

Optical Characterization. Differential reflectance spectra were acquired with a home-built microreflectance setup. [1,2] Measurements were taken from a ∼1.4 μm spot using a Thorlabs CCS200/M fiber-coupled spectrometer (Thorlabs, Inc., Newton, New Jersey, U.S.A.) and a Motic BA310 MET-T microscope equipped with a 50× objective and an AMScope MU1803 CMOS camera.

Raman measurements were performed using a MonoVista CRS+ system (Spectroscopy and Imaging GmbH) with 532 nm excitation, a 50× objective, and a 2400 lines/mm diffraction grating. Laser power was kept below 0.3 mW to avoid heating or damaging the flakes. The spectra were collected with a 60 s integration time. A photograph of the straining setup used for collecting the Raman data is shown in the next section (Figure S1).

Straining Setup. A home-built cruciform bending apparatus was used to apply biaxial strain to the samples. Strain calibration was performed using patterned micropillars, which enabled direct measurement of the applied strain. Further details of the setup are provided in the study by Carrascoso et al[3].

S2. Biaxial straining setup

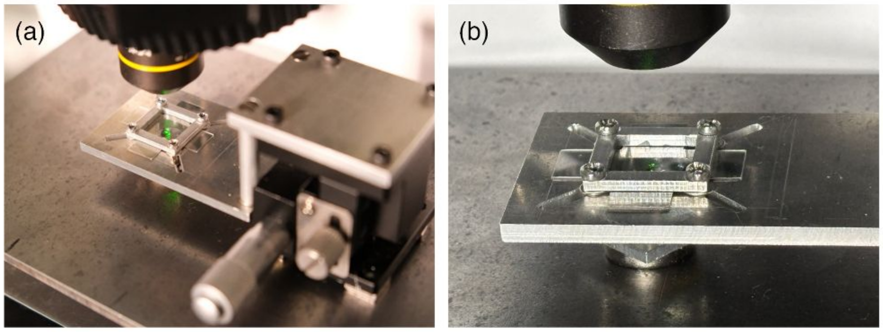

Figure S1. Experimental setup for applying biaxial strain under the Raman microscope. Schematic of the cruciform bending platform used to impose isotropic in-plane tensile strain on WS_2 flakes while performing Raman measurements. The sample is mounted at the center of the cruciform, and the vertical displacement of the central Z-stage produces a uniform biaxial expansion in the central region. The configuration enables simultaneous collection of Raman spectra with 50× objective while controlling and calibrating the applied strain.

S3. Identification of number of layers

To confirm the layer number of the WS_2 flakes used in this study, atomic force microscopy (AFM) measurements were performed on regions containing monolayer (1L), bilayer (2L), and trilayer (3L) areas (Figure S2). Because the thickness of individual WS_2 layers is below 1 nm, absolute height values obtained by AFM can depend on tip-sample interactions, surface adsorbates, and substrate effects. Therefore, AFM was primarily used to identify relative step-height increments between adjacent regions rather than to extract absolute thickness values. Layer assignment was further supported by the Raman spectra acquired from the same regions (Figure S2(b)).

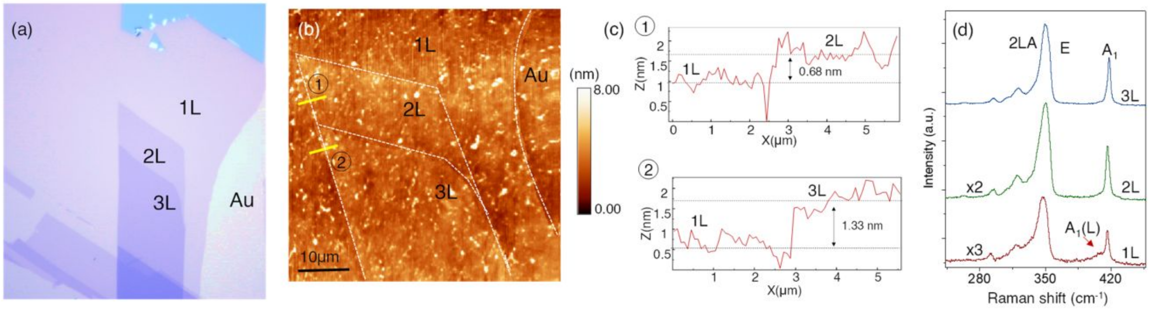

Figure S2. Layer identification of WS_2 flakes. (a) Optical micrograph showing monolayer (1L), bilayer (2L), and trilayer (3L) regions. (b) AFM height image and (c) line profiles showing the relative step-height differences between adjacent regions. (d) Raman spectra acquired from each region.

S4. Raman fits for monolayer and bilayer WS_2

We analyzed the Raman spectra of monolayer and bilayer WS_2 on Au. Consistent with previous observations for WS_2 on Au,[4] a low-frequency A_1 (L) mode appears in the monolayer at 417.5 cm^{-1}. Figure S3 displays the experimental Raman spectra together with the multi-peak Voigt fits used for the A_1, E, and 2LA(M) modes, as well as the evolution of their peak positions as a function of biaxial strain. As expected, both the in-plane and out-of-plane phonons soften with increasing strain for monolayers and bilayers. The collapse of the 2LA(M) mode is also observed, although it becomes more pronounced in the trilayer. In the monolayer, the strain-induced shifts remain smaller than those measured in the bilayer and

trilayer. These results confirm that the observed phonon shifts and the strain-induced suppression of the 2LA(M) band are robust and not artifacts of fitting instability.

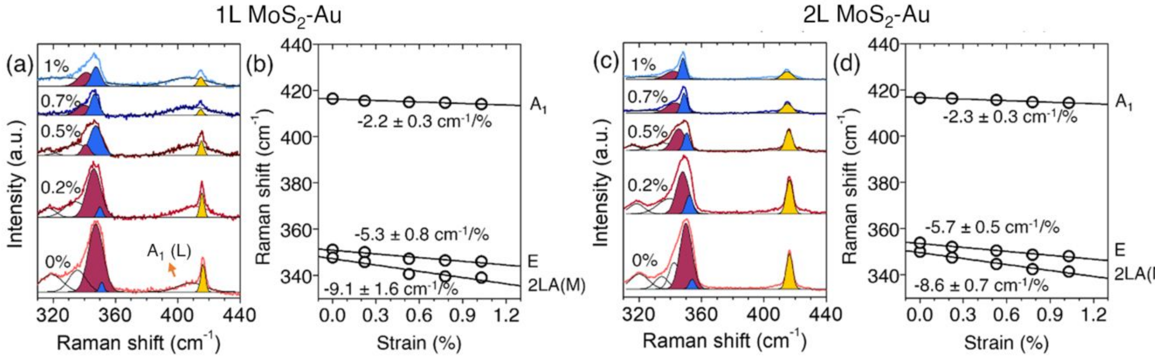

Figure S3. Raman analysis of monolayer (1L) and bilayer (2L) WS_2 on Au under biaxial strain. (a) Experimental Raman spectra of monolayer WS_2 (black curves) together with multi-peak Voigt fits for the E, A_1, and 2LA(M) modes (colored components). (b) Evolution of the fitted peak positions as a function of applied strain. The in-plane (E) and out-of-plane (A_1) phonons soften with increasing strain, and the 2LA(M) mode exhibits a progressive intensity collapse. (c) Voigt fits for the E, A_1, and 2LA(M) modes for bilayer WS_2. (d) Strain-dependent frequency shifts, showing phonon softening with increasing strain.

The Grüneisen parameter (γ) was obtained from the biaxial strain dependence of the Raman frequencies using $\frac{\Delta\omega}{\omega_0} = -2\gamma\varepsilon$, which leads to $\gamma = -\frac{1}{2\omega_0}\frac{d\omega}{d\varepsilon}$.

Grüneisen parameter (γ)

Raman mode	Monolayer WS_2-Au	Bilayer WS_2-Au	Trilayer WS_2-Au
**A_1**	0.26	0.28	0.28
**E**	0.75	0.81	0.96
**2LA(M)**	1.32	1.23	1.54

Table S1. Grüneisen parameters for the Raman modes of monolayer, bilayer and trilayer WS_2. Values were obtained from the biaxial strain dependence of the phonon frequencies.

S5. Background subtraction procedure for ΔR/R spectra

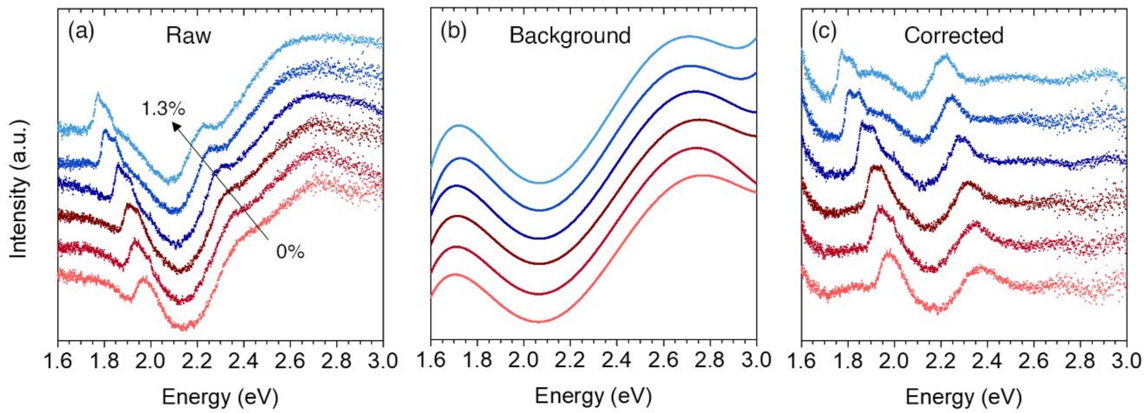

Figure S4. Representative example of the background subtraction procedure applied to ΔR/R spectra. (a) Raw differential reflectance spectrum under applied biaxial strain. (b) Background function used in the subtraction. (c) Background-corrected spectrum used for exciton peak fitting and energy extraction.

S6. Spatial uniformity of the applied biaxial strain

To assess the spatial uniformity of the applied biaxial strain, a Raman map (Figure S5) was acquired over a 100 × 100 μm^2 region of a few-layer WS_2 flake under an applied strain of approximately 0.6%. The local strain was calculated from the E-mode frequency using the experimentally determined gauge factor for trilayer WS_2.

The strain map reveals minimal spatial variation across the scanned area. The histogram of extracted strain values shows a narrow distribution centered at ~0.6%, with a standard deviation of 0.09%, corresponding to less than 0.1% absolute strain variation. These results confirm that the strain field in the central region of the cruciform platform is spatially homogeneous within the resolution of the Raman measurement.

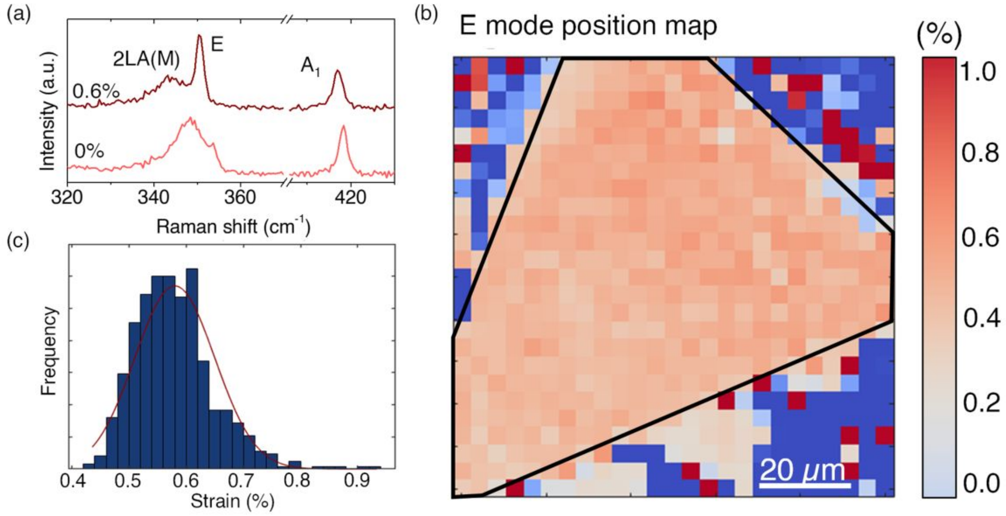

Figure S5. Spatial uniformity of the applied biaxial strain. (a) Raman spectra of a WS_2 flake recorded before strain application (0%) and under applied biaxial strain (~0.6%), showing the redshift of the E mode used for strain quantification. (b) Spatial Raman map (100 ×100 μm^2) of the extracted biaxial strain, calculated from the local E-mode frequency using the experimentally determined gauge factor for trilayer WS_2 and the unstrained reference position. (c) Histogram of the strain values extracted from the Raman map, showing a narrow distribution centered on the nominal applied strain.

S7. Reversibility and absence of hysteresis under biaxial strain

To ensure that the WS_2 flakes undergo purely elastic deformation under biaxial loading, we performed controlled loading–unloading experiments over multiple strain cycles. Figure S6(a) shows the Raman spectra acquired while the applied strain was gradually increased and subsequently reduced back to zero. The E, A_1, and 2LA(M) peak positions return to their initial unstrained values within experimental uncertainty, indicating full recovery of the phonon frequencies. Figure S6(b) shows the evolution of the phonon peak positions over repeated loading-unloading cycles between 0 and 1% strain. No measurable offset between loading and unloading trajectories is observed, demonstrating the absence of detectable hysteresis.

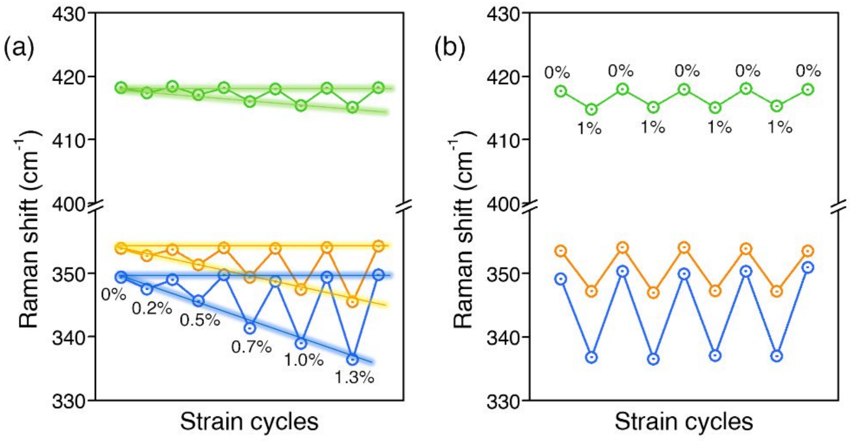

Figure S6. Reversibility of applied biaxial strain in trilayer WS_2. (a) A_1, E, and 2LA(M) peak positions when strain is increased in small increments and subsequently released back to the unstrained state. (b) A_1, E, and 2LA(M) peak positions during repeated strain cycles between 0 and 1%.

To further assess mechanical stability, repeated strain cycles between 0 and 1% were performed. Representative Raman spectra collected over multiple (>10) loading–unloading cycles are shown in Figure S7. The peak positions remain consistent across cycles, with no spectral broadening, peak splitting, or irreversible frequency shifts. These results rule out slippage, plastic deformation, or sample degradation.

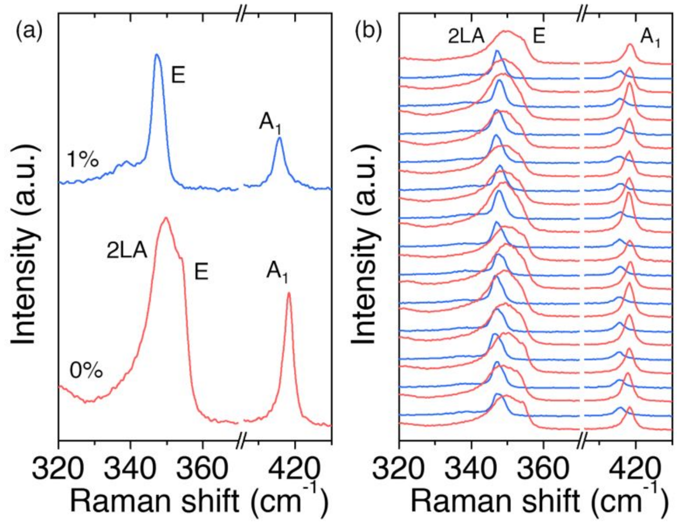

Figure S7. Multiple strain cycles. (a) Raman spectra acquired over more than 10 loading-unloading cycles between 0 and 1% biaxial strain, demonstrating reproducible and reversible peak shifts.

S8. Double-resonant Raman model and effective detuning description

The 2LA(M) mode in WS_2 arises from a two-phonon double-resonant process in which the scattering amplitude is mediated by intermediate electronic states. In a rigorous description, this process is treated within fourth-order time-dependent perturbation theory, where the scattering amplitude contains multiple resonance denominators associated with intermediate electronic or excitonic states and explicitly includes the phonon energies involved in the process. A commonly used schematic form for the intensity can be written as[5,6]:

$$I \propto \left| \sum_{\mathrm{k}} \frac{M}{(E_L - E_1(\mathrm{k}) + i\gamma_1)\,(E_L - E_2(\mathrm{k}) - \hbar\omega_q + i\gamma_2)\,(E_L - E_3(\mathrm{k}) - \hbar\omega_q - \hbar\omega_{-q} + i\gamma_3)} \right|^2$$

where E_L is the laser energy, $E_1(\mathrm{k})$, $E_2(\mathrm{k})$, $E_3(\mathrm{k})$ are intermediate electronic or excitonic energies along the scattering pathway, $\hbar\omega_{\pm q}$ are the phonon energies involved in the two phonon process, $i\gamma_{\mathrm{i}}$ are damping terms, and M collects optical and electron phonon matrix elements.

This expression explicitly shows that the resonance condition depends both on the excitonic intermediate energies and on the phonon energies entering the denominators.

S8.1. Reduction to an exciton-dominated detuning model

In the present experiment, biaxial strain primarily shifts the excitonic transition energy, while the LA phonon energies vary much more weakly over the same strain range. Importantly, the phonon energies are not negligible in the resonance condition because $2\hbar\omega_{LA}$ is on the order of tens of meV. What is small is their strain-induced variation compared to the excitonic redshift.

From our data, the B exciton shifts by approximately 138 meV/% strain, while the 2LA(M) peak position shifts by 1.34 meV/% strain. Over the full strain range of ε_{max}=1.3, this corresponds to a change of E_{Xb} of 180 meV, while $2\hbar\omega_{LA}$ changes by only 1.7 meV. This large difference in scale justifies modeling the strain dependence of the double-resonant enhancement primarily through the strain-dependent exciton-laser detuning.

Under these conditions, the dominant strain-dependent term in the resonance denominators is the excitonic energy. The full multi-denominator expression can therefore be reduced to an effective detuning form that captures the experimentally observed evolution of the resonance window.

S8.2. Effective resonance description

Within this reduced description, the Raman amplitude can be expressed in a semiclassical Kramers–Heisenberg formalism for an exciton-assisted process as[7,8]

$$R \propto \frac{\langle f|H_{ep}|n\rangle \langle n|H_{e-p}|i\rangle}{(E_L - E_x + i\Gamma_X)}$$

where E_x is the exciton energy, Γ_X is the intrinsic exciton linewidth. The finite k-space extension of the double-resonant pathway further broadens the resonance beyond the intrinsic exciton width. We therefore introduce an effective width:

$$\Gamma_{\mathrm{eff}}{}^2 = \Gamma_X^2 + \Gamma_{\mathrm{DR}}^2 + \Gamma_{\mathrm{ph}}^2$$

where Γ_{DR} accounts for the range of off-shell electronic states participating in the double-resonant pathway, and Γ_{ph} reflects the finite dispersion of LA phonons near M.

To make the phonon contribution explicit while retaining a compact fit form, the intensity of the 2LA(M) mode is written as:

$$I(\varepsilon) = \frac{A}{(E_L - E_{\mathrm{Xb}}(\varepsilon) - 2\hbar\omega_{LA} - \delta_0)^2 + \Gamma_{\mathrm{eff}}{}^2}$$

where $2\hbar\omega_{LA}$ is taken from the measured 2LA(M) Raman shift at each strain value, and δ_0 accounts for the residual offset between the measured B exciton peak energy and the effective intermediate energy entering the double resonant pathway.

In this representation, the parameter used in the main text is recovered through the definition, $\Delta_0 = 2\hbar\omega_{LA} + \delta_0$, which separates the known two-phonon energy from the remaining

effective detuning parameter. The parameters extracted from the fit (see main text, Fig. 5c) quantify the effective resonance window explored in the double-resonant process.

REFERENCES

(1) Castellanos-Gomez, A. Retrofitting a Commercial Upright Microscope to Perform Micro-Transmittance/Reflectance Spectroscopy. 2023. https://doi.org/10.5281/zenodo.7525335.

(2) Frisenda, R.; Drüppel, M.; Schmidt, R.; Michaelis de Vasconcellos, S.; Perez de Lara, D.; Bratschitsch, R.; Rohlfing, M.; Castellanos-Gomez, A. Biaxial Strain Tuning of the Optical Properties of Single-Layer Transition Metal Dichalcogenides. *npj 2D Mater. Appl.* **2017**, *1* (1), 10. https://doi.org/10.1038/s41699-017-0013-7.

(3) Carrascoso, F.; Frisenda, R.; Castellanos-Gomez, A. Biaxial versus Uniaxial Strain Tuning of Single-Layer MoS_2. *Nano Mater. Sci.* **2022**, *4* (1), 44–51. https://doi.org/https://doi.org/10.1016/j.nanoms.2021.03.001.

(4) Rodriguez, A.; Velický, M.; Řáhová, J.; Zólyomi, V.; Koltai, J.; Kalbáč, M.; Frank, O. Activation of Raman Modes in Monolayer Transition Metal Dichalcogenides through Strong Interaction with Gold. *Phys. Rev. B* **2022**, *105* (19), 195413. https://doi.org/10.1103/PhysRevB.105.195413.

(5) Carvalho, B. R.; Wang, Y.; Mignuzzi, S.; Roy, D.; Terrones, M.; Fantini, C.; Crespi, V. H.; Malard, L. M.; Pimenta, M. A. Intervalley Scattering by Acoustic Phonons in Two-Dimensional MoS_2 Revealed by Double-Resonance Raman Spectroscopy. *Nat. Commun.* **2017**, *8* (1), 14670. https://doi.org/10.1038/ncomms14670.

(6) Lemos, J. S.; Blundo, E.; Polimeni, A.; Pimenta, M. A.; Righi, A. Exciton–Phonon Interactions in Strained Domes of Monolayer MoS_2 Studied by Resonance Raman Spectroscopy. *Nanomaterials*. 2023, p 2722. https://doi.org/10.3390/nano13192722.

(7) Livneh, T. Resonant Raman Scattering in UO_2 Revisited. *Phys. Rev. B* **2022**, *105* (4), 45115. https://doi.org/10.1103/PhysRevB.105.045115.

(8) Vamivakas, A. N.; Walsh, A.; Yin, Y.; Ünlü, M. S.; Goldberg, B. B.; Swan, A. K. Exciton-Mediated One-Phonon Resonant Raman Scattering from One-Dimensional Systems. *Phys. Rev. B* **2006**, *74* (20), 205405. https://doi.org/10.1103/PhysRevB.74.205405.
